# Analysis of the 4q35 chromatin organization reveals distinct long-range interactions in patients affected with Facio-Scapulo-Humeral Dystrophy

**DOI:** 10.1038/s41598-019-46861-x

**Published:** 2019-07-17

**Authors:** Marie-Cécile Gaillard, Natacha Broucqsault, Julia Morere, Camille Laberthonnière, Camille Dion, Cherif Badja, Stéphane Roche, Karine Nguyen, Frédérique Magdinier, Jérôme D. Robin

**Affiliations:** 1 Aix Marseille Univ, INSERM, MMG, U 1251 Marseille, France; 2 APHM, Laboratoire de Génétique Médicale, Hôpital de la Timone, Marseille, France

**Keywords:** Diseases, Pathogenesis, Epigenetics, Chromatin structure

## Abstract

Facio-Scapulo Humeral dystrophy (FSHD) is the third most common myopathy, affecting 1 amongst 10,000 individuals (FSHD1, OMIM #158900). This autosomal dominant pathology is associated in 95% of cases with genetic and epigenetic alterations in the subtelomeric region at the extremity of the long arm of chromosome 4 (q arm). A large proportion of the remaining 5% of cases carry a mutation in the *SMCHD1* gene (FSHD2, OMIM #158901). Here, we explored the 3D organization of the 4q35 locus by three-dimensions DNA *in situ* fluorescent hybridization (3D-FISH) in primary fibroblasts isolated from patients and healthy donors. We found that D4Z4 contractions and/or *SMCHD1* mutations impact the spatial organization of the 4q35 region and trigger changes in the expression of different genes. Changes in gene expression were corroborated in muscle biopsies suggesting that the modified chromatin landscape impelled a modulation in the level of expression of a number of genes across the 4q35 locus in FSHD. Using induced pluripotent stem cells (hIPSC), we further examined whether chromatin organization is inherited after reprogramming or acquired during differentiation and showed that folding of the 4q35 region is modified upon differentiation. These results together with previous findings highlight the role of the D4Z4 macrosatellite repeat in the topological organization of chromatin and further indicate that the D4Z4-dependent 3D structure induces transcriptional changes of 4q35 genes expression.

## Introduction

Over the recent years, sequence distribution within the nuclear space, nuclear topology and long-distance interactions have emerged as key elements in the regulation of gene expression and cell fate determination. The genome is separated from the cytoplasm by the nuclear envelope. The inner nuclear membrane is composed of specialized proteins, which contribute to higher-order organization of the genome within the nuclear space and the regulation of gene expression, with repressed loci mainly located at the nuclear periphery^1,2^. Interphase chromosomes reside in minimally overlapping chromosome territories with active genes mainly oriented toward the nuclear interior^3^. Individual genes are largely confined to their respective chromosome territories (CT). However, in certain developmental contexts, certain loci such as the *Hox* genes or genes on the inactive X chromosomes can loop out of their CTs^4,5^. Furthermore, long distance dynamic interactions between sequences or subnuclear domains have been identified by various genome-wide approaches^6–8^. Thus, different topological levels shape the human genome, from large scale folding involving *cis-* or *trans*- long-distance interactions to smaller interactions between regulatory elements located in close proximity^9^. Interestingly, topological conformation is highly conserved between cell types and species suggesting that it is sequence-dependent rather that controlled by cell differentiation processes^10,11^. However, so far only a few sequences with topological activity have been identified and characterized^12–14^. Amongst them, repetitive elements involved in pathologies such as expanded triplets^15^ or the D4Z4 macrosatellite^12,13,16^ have been shown to cause topological modifications.

Regarding this disease-associated topological disorganization, one intriguing locus is the 4q35 subtelomere^17,18^. This gene-poor locus characterized by the presence of gene deserts and large blocks of repetitive DNA sequence, including blocks of (CA)_n_ microsatellites, variable number of tandem 3.3 Kb D4Z4 macrosatellite repeats and β satellite elements is localized at the nuclear periphery, a nuclear compartment enriched in heterochromatin^12,17–20^. The 4q35 genes are organized in clusters separated by domains associated with the nuclear lamina (Lamin Associated Domains, LAD) and limited by CTCF-dependent boundaries^21,22^.

We have previously described the D4Z4 macrosatellite as the only known element able to tether any telomere at the nuclear periphery and control the replication timing of its abutting telomeric region. As usually observed for heterochromatin-rich regions, the 4q35 telomere replicates late, at the end of the S phase^23^ but displays features of repressed euchromatin rather than constitutive heterochromatin^24^.

Shortening of the D4Z4 array at the 4q35 locus is linked to Facio-Scapulo-Humeral Dystrophy (FSHD), the third most common hereditary myopathy. In 95% of cases, FSHD is linked to the deletion of an integral number of D4Z4, on one of the two 4q35 alleles^25,26^. Affected individuals exhibit between 1–10 repeats whereas the general population have a number of repeats above 10 (up to 100)^27^. Shortening of this repetitive the array is associated with DNA hypomethylation and relaxation of the macrosatellite chromatin suggesting the involvement of epigenetic changes in the disease^16,28–30^. In agreement, the remaining 5% of patients usually display a profound hypomethylation and 80% of them carry a mutation in gene encoding the SMCHD1 (Structural Maintenance of Chromosomal Hinge Domain Containing 1) protein^31^. In mice, *Smchd1* loss of function results in early lethality for female embryos, attributed to derepression of genes on the inactive X chromosome^32^. Smchd1 is also involved in silencing of repetitive DNA sequences and formation of long distance loops at the *Hox* genes loci and inactive X chromosome^33^.

As a consequence of the D4Z4 array hypomethylation, the current patho-physiological model for FSHD implicates the ectopic expression of *DUX4*, a retrogene present in each D4Z4 unit but whose protein is encoded by the last D4Z4 and downstream sequence. In this scheme, functional *DUX4* transcripts are produced by the last unit and stabilized in individuals carrying a permissive 4qA haplotype, that includes a poly adenylation signal (pLAM) in the direct proximity of the last D4Z4 unit. Three subtelomeric 4q35 haplotypes have been identified (A, B, C), with A and B being predominant and equally distributed in the general population^34^. Of note, D4Z4 units are also found at other loci such as the short arm of acrocentric chromosomes. In particular, the subtelomeric 10q locus shares 98% homology with the 4q35 locus but is not associated with FSHD.

By using a three-dimensional Fluorescent *In Situ* Hybridization approach (3D FISH), we have previously shown the existence of functional interactions between D4Z4, the nuclear lamina and the telomere^12,13,16,23^ with a key role for D4Z4 in the organization and regulation of long-distance interactions at this locus. These observations were further corroborated by chromatin conformation capture approaches that revealed the influence of the contracted units on the 3D organization with chromatin loops encompassing domains beyond the D4Z4 units^35–37^. The higher-order regulation of this region is also modulated by telomere length^16^, suggesting possible cooperation between different specialized genomic elements in the topological organization of this region. Hence, partitioning of the 4q35 region into different topological domains raises the question of the regulation of long-distance interactions across the locus and the role of the D4Z4 array in this higher-order organization.

In order to get a precise insight into the 3D DNA interactions within the 4q35 locus in normal and pathological conditions, *i.e*., influence of D4Z4 array size or *SMCHD1* mutation, we investigated the three-dimensional chromatin organization of the locus using hybridization techniques in primary cells isolated from FSHD individuals and controls. We identified different loops organizing the 4q35 locus into different chromatin domains. In agreement with previous findings and a role for D4Z4 in chromatin topology, these chromatin domains are modified when the number of D4Z4 units is decreased. Moreover, we analyzed expression of a number of genes localized within or outside of these loops in our cells and confronted our results to those obtained in muscle biopsies from affected and unaffected individuals.

## Results

### Exploring the 3D organization of the 4q35 locus

Given the peculiar organization of the 4q35 region and the topological role played by D4Z4 in healthy and diseased cells^12,13,16^, we aimed at deciphering the higher-order changes of this locus in FSHD1 and 2 patients compared to healthy donors. To this aim, we first analyzed datasets available in the literature^6^. Indeed, In the past decade, a wealth of chromatin techniques coupled to deep-sequencing technologies have emerged and multiple databases for 3D DNA organization are available^11,38,39^. From these, we retrieved 4q35 locus interaction maps (183–191 Mb of Chr. 4) generated from 3 different cell lines and 1 muscle biopsy (Supplementary Fig. 1). Amongst these maps, two achieved a Kb resolution (GM12878, IMR90) while the remaining two barely achieved a Mb resolution (STL002, *Psoas* muscle biopsy), a poor resolution mostly due to the depth of sequencing, number of assays performed and nature of the samples.

Altogether these maps obtained from different cell types show a conserved domain organization for the most subtelomeric part of the locus up to a region located between the *FAT1* and *SORBS2* genes (187 and 186 Mb of Chr.4; respectively), two genes previously implicated in FSHD^16,40,41^. When observed at a Mb level, these maps suggest that the chromatin organization and domains (TADs) are set, independently of cell types and tissue of origin, *i.e*., conserved between lymphocytes, fibroblasts and muscle (*Psoas*).

Given the involvement of genomic rearrangement of this subtelomeric region in FSHD, we next set out to investigate the 4q35 chromatin organization by 3D DNA FISH using selected probes in well-defined cells (Table 1). Unlike deep sequencing techniques such as chromatin conformation capture (3C, HiC) or replication timing assays, this low-resolution approach with a limitation of resolution of approximately 300Kb has the unique advantage of allowing the analysis of each individual allele at a single cell level. To further investigate whether D4Z4 array shortening below the 11-units threshold is associated to 4q35 topological changes, we used primary fibroblasts isolated from skin biopsies from five different FSHD1 patients, one of which presenting a homozygous D4Z4 contraction (*i.e*., 8 UR) along with fibroblasts isolated from two FSHD2 individuals (*i.e*., >10 UR) carrier of a mutation in *SMCHD1* (Table 1) or healthy donors.

**Table 1:** Summary of cells (fibroblasts from skin biopsies) used in the study with their respective associated status and number of D4Z4 repeats (RU) at the 4q and 10q locus along with mutation found in *SMCHD1*.

Sample	Status	4q	10q	Age	Sex	hIPSCs	SMCHD1
A1	Control	>10RU	NA	Foetus	♀	Available	WT
A2 (ID: AG08498)	Control	>10RU	NA	1	♂		WT
A3	Control	>20RU	NA	21	♂		WT
N (ID: 12759)	FSHD1	7RU qA > 10RU qB	NA	51	♀	Available	WT
M (ID: 12566)	FSHD1	3.5RU qA 22RU qB	NA	54	♀		WT
F (ID: 120723)	FSHD1	6RU qA > 10RU qB	NA	28	♀		WT
T (ID: TalF)	FSHD1	2RU qA > 10RU qB	NA	13	♂		WT
H	FSHD1	8RU qA 8RU qA	NA	29	♂		WT
G (ID: 11440)	FSHD2	12RU qA 32RU qA	14UR qA 17UR qA	37	♂		c.2338 + 4A > G intron 18 r.2261_2337del; p.S754*
S (ID: 15166)	FSHD2	12RU qA 23RU qB	13UR qB 20UR qB	67	♀		c.4614_4615 insTATAATA; r.4614_4615 insTATAATA; p.A1539Yfs*4

FSHD individuals were diagnosed by clinical examination by neurologists with clinical expertise in the evaluation of neuromuscular diseases. The genetic status was confirmed by genetic testing (Southern Blot, DNA combing). Gender and age at biopsy are indicated as well as the corresponding identification number (ID) of samples used elsewhere^56^.

**Figure 1:**
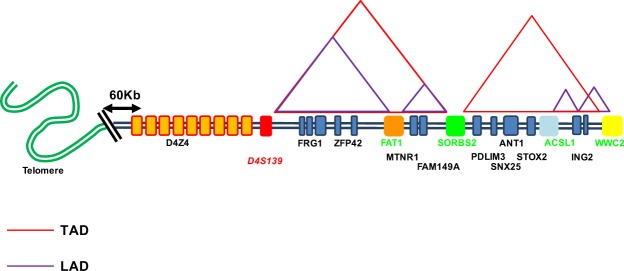
Organization of the 4q35 region. Graphical representation of the subtelomeric region of chromosome 4 (4q35 locus) from the telomere to the *WWC2* gene. Using available data from the literature (*i.e*., HiC, ChIA-PET) we report the detected TADs (Red) and LADs (purple) found and preserved across cell types.

As no clear TADs were found in more centromeric regions and given the putative interactions between the FSHD locus and the nuclear lamina or matrix^12,16–18,37^, we focused our attention on the different regions separated by LADs^22^ and the conserved TAD revealed by available deep sequencing datasets^11,39,42^ (Fig. 1). The 4q35 locus contains four LADs. The most distal one (190–191 Mb of Chr 4) is located upstream of the D4Z4 array and overlaps with a large gene-desert between the region containing the *ZFP42* and *FAT1* genes. The second LAD (187–187.7 Mb) separates the *FAT1* gene from the *SORBS2* region and encompasses several genes (including *MTNR1A* and *FAM149A*). The third (184.8–185.4 Mb) and fourth (185.6–185.8 Mb) LADs are situated in a gene-poor region, suggesting the existence of two loops anchored at the nuclear periphery between the *ACSL1* and *WWC2*-containing domains^43^. Interestingly, the *WWC2* region correspond to a TAD boundary in at least two data sets (GM12878, IMR90; respectively)^6^. To understand higher-order 3D folding patterns of the 4q35 region in the different contexts, we selected five probes encompassing the whole 4q35 regions and the different putative topological domains (Fig. 1).

### 3D genome organization of the 4q35 locus is modified in FSHD

Chromatin Conformation Capture based analysis (chromatin studies in cell populations, *e.g*. HiC) reveal a global conservation of genomic domains, regardless of the cell types. Considering the cell-by-cell resolution of FISH-based techniques and taking advantage of the conserved chromatin topology between cell types and tissues, we investigated the three-dimensional organization of the 4q35 region of each individual allele *i.e*., healthy and diseased alleles in primary fibroblasts from FSHD patients and healthy donors^42,44^. FSHD patients have all been carefully diagnosed at the clinical level by experienced neurologists and at the molecular level by DNA combing^45,46^ (Table 1). For FSHD1, we used cases with a range of contracted D4Z4 arrays restricted to the 4qA-type haplotype (2, 4, 6, 7 and 8 Repeated Units (RU); respectively) representative of the heterogeneity seen in FSHD1. This group includes cells from a patient carrying two contracted 4qA allele (H). Likewise, D4Z4 repeats were >10RU in FSHD2 individuals who are carriers of a mutation in the *SMCHD1* gene.

**Figure 2:**
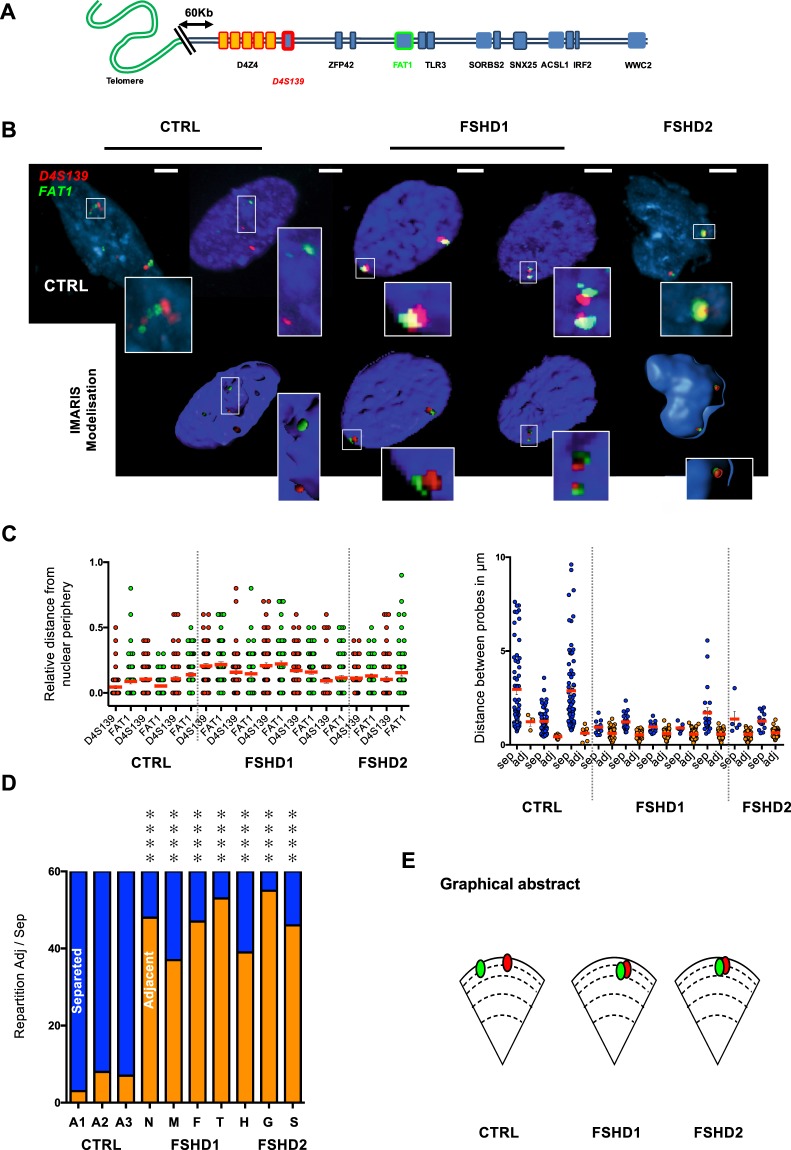
The distance between the distal 4q35 region and the *FAT1* gene is decreased in cells (fibroblasts) from FSHD1 and 2 patients. (**A**) Graphical representation of the subtelomeric region of chromosome 4, from the telomere (green) to the *WWC2* gene (blue square) located at a distance of 7 Mb from the D4Z4 array. 3D FISH was performed using two sets of probes corresponding either to the D4S139 variable number tandem minisatellite (VNTR; red) or the *FAT1* region (green). (**B**) Representative pictures of FISH images in primary fibroblasts from healthy donors (control) or FSHD1 and FSHD2 patients and 3D reconstruction using IMARIS. Scale is indicated by a white bar (=2 µm). (**C**) Quantification of FISH signal was done using IMARIS. We measured the distance of each probe to the nuclear periphery (left) and distance between the probes (right). At least 30 nuclei were analyzed (60 alleles). For each cell, we evaluated if signals were separated or adjacent (sep, adj; respectively). (**D**) Summary of events and associated Chi-Square test (n = 30 cells per sample; 60 alleles). Frequency of adjacent signals is increased in all FSHD cells. ****p < 0.001. (**E**) Summary of the average distances between probes and their respective distances to the periphery in each sample. Signals are mostly localized at the periphery regardless of disease status.

**Figure 3:**
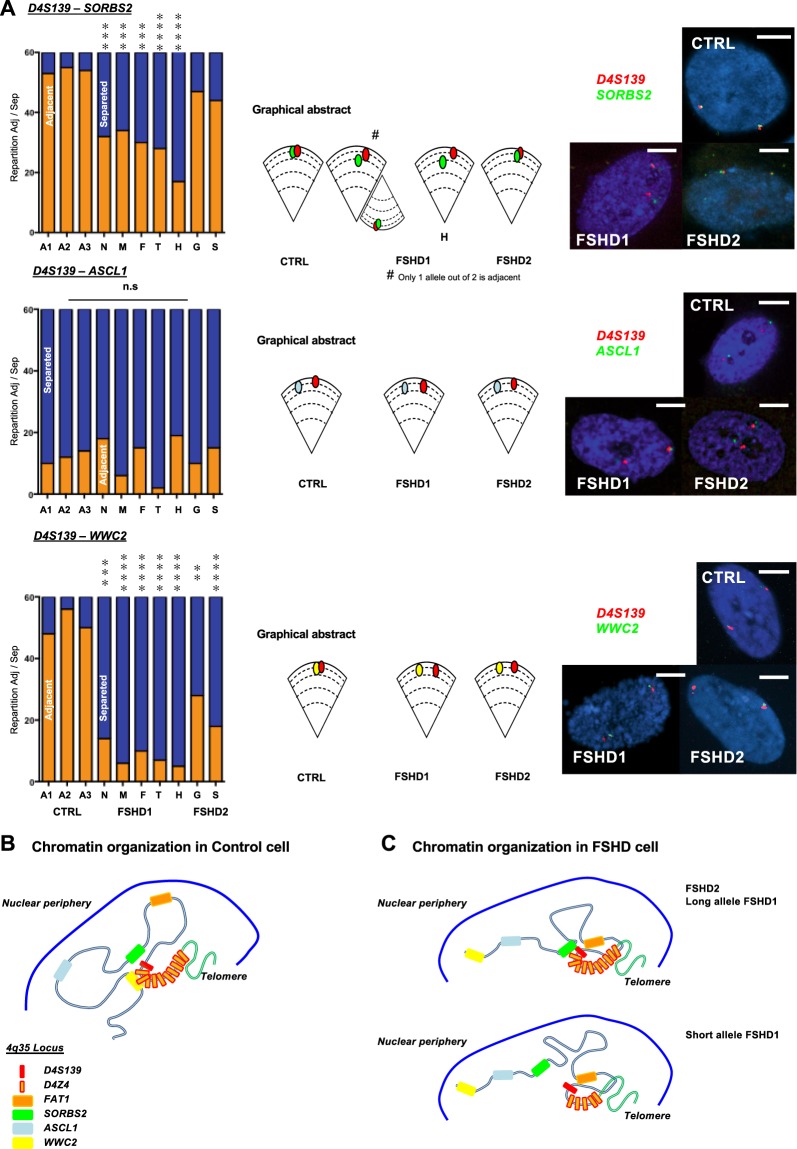
Distinct long distance interactions at the 4q35 region in controls and FSHD cells (fibroblasts). (**A**) Quantifications of colocalization and nuclear distribution of 3D DNA FISH assay within the 4q35 locus. We report the frequency of association between the D4S139 region (red; as in Fig. 2) and either the *SORBS2*, *ASCL1* or *WWC2* region (green, blue, yellow; respectively) in primary fibroblasts from healthy donors, FSHD1 or FSHD2 patients. A Chi-Square test was performed for each assay to determine statistical differences (n = 30 cells per sample, 60 alleles). (**B**,**C**) Graphical representation of the 4q35 chromatin organization as revealed by 3D DNA FISH in control cells (**B**) and FSHD1 and 2 cells (**C**). **p < 0.01; ***p < 0.005; ****p < 0.001.

We performed 3D DNA FISH in FSHD1 (n = 5), FSHD2 (n = 2) and control (n = 3) cells, using for each assay two sets of probes (Figs 2 and 3, Supplementary Figs 2–5). One probe was common between all experiments and hybridizes with the D4S139 minisatellite (red) proximal to the D4Z4 repetitive array and corresponding to the most proximal region specific to the 4q35 locus (Fig. 2A). Rationale for the choice of remaining probes is described above and targeted the *FAT1*, *SORBS2*, *ASCL1* and *WWC2* regions, respectively (green). After hybridization and confocal imaging, stacks of nuclei were processed using the IMARIS software (Bitplane, AG) for 3D reconstruction and analysis (n > 30 nuclei per sample and per condition; *i.e*., 60 alleles) (Fig. 2B, Supplementary Fig. 2). Three Dimensions computational modeling allows one to retrieve accurate values from confocal imaging such as volume of nuclei and probes, spatial distribution, position (relative to the nuclear envelope) and distances between probes (Fig. 2C). We compiled results for each assay (4 assays; >300 nuclei analyzed per assay) and asked whether global 4q35 folding is identical between cells from controls and patients and between FSHD1 and FSHD2.

First, all signals were found at the nuclear periphery regardless of disease status and D4Z4 contractions (average distance from nuclear periphery < 0.3 µm; Figs 1 and 2) indicating that the size of the D4Z4 array does not modify the radial position of the locus within the nucleus. Next, we evaluated if signals between probes were separated or adjacent (noted sep or adj; respectively). This binary decision (sep, adj) was based on physical observations of separated or interacting objects (determined by IMARIS) and measurement of distances between the gravity center of the probe signal after 3D reconstruction (Supplementary Fig. 2). Overall, distances associated to adjacent and separated events were significantly different between conditions (*p* < 0.0001; Mann Whitney, *α* = 0.05). All assays considered (n = 1400 nuclei; 2800 signals) we measured a mean distance of 0.73 µm (±SD = 0.23 µm) for adjacent signals and 3.92 µm (±SD = 1.43 µm) for separated signals.

In control cells, the mean distance between the D4S139 region and *FAT1* was significantly higher than in FSHD cells (Fig. 2C, Mean ± SD; CTRL = 2.26 ± SD = 0.98 µm and FSHD = 0.73 ± SD = 0.24 µm; *p* < 0.0001; Mann-Whitney, *α* = 0.05). Accordingly, signals were mostly separated in all control cells (90%) and adjacent in FSHD cells (Fig. 2D, FSHD1 = 75%; FSHD2 = 84%). Since both alleles behaved similarly (95%) regardless of number of repeats, our data suggest a modulation of the chromatin structure independent of the D4Z4 array (Fig. 2E).

Strikingly, regarding *SORBS2*, we observed a partial loss of interaction restricted to FSHD1 cells. Indeed, when considering FSHD1 cells carrying only one contracted allele (Table 1, N, M, F and T), 90% of cells displayed a partial loss of interaction where only one out of two signals, *i.e*., allele, were separated (Fig. 3A). Accordingly, in FSDH1 cells carrying two contracted alleles (Table 1, H), the interaction was completely lost (Fig. 3, *p* < 0.001; Chi-Square test, α = 0.05) suggesting that this interaction is alleviated when the number of D4Z4 repeats is decreased as previously observed^16^. In FSHD2 cells (Table 1, G and S), the interaction was preserved but a subset of cells (24%) displayed a complete loss of interaction (for both alleles) as opposed to a smaller subset in control cells (10%). This suggest a dynamic interaction influenced by other parameters (*e.g*., SMCHD1 status, DNA methylation, telomere length).

Next, regarding the *ASCL1* region, no difference was found between samples considering the D4S139 and *ASCL1* probes (Mean ± SD; CTRL = 3.91 ± SD = 1.38 µm and FSHD = 4.1 ± SD = 1.7 µm; *p* = 0.97; Mann-Whitney, α = 0.05), for which signals were separated in all situations, further suggesting that compaction of chromatin was similar between all samples analyzed.

Last, we looked at the *WWC2* region and found a majority of adjacent signals (85%) with a probe targeting the D4S139 region, in control cells (Mean ± SD; CTRL = 0.66 ± SD = 0.22 µm). Oppositely, probe signals were separated in most FSHD cells (62%) regardless of the number of D4Z4 repeats.

Altogether, our data show that in primary FSHD fibroblast, the D4S139 region no longer interacts with *WWC2* but with *FAT1* (*p* < 0.001 for FSHD cases; Chi-Square test, α = 0.05). In control cells, the D4S139 region is localized in the vicinity of the *SORBS2* and *WWC2* regions, but separated from *FAT1* and *ASCL1* (all in different LADs), a finding reminiscent of the TAD identified in the literature (Fig. 1, Supplementary Fig. 1)^6^. In FSHD cells, interactions were identical between FSHD1 and 2 in all conditions but one (D4S139-*SORBS2*; Fig. 3A). This suggests that the topological organization of this locus is orchestrated by epigenetic features associated with the D4Z4 array, either in -*cis* (number of repeats for FSHD1) or -*trans* (*SMCHD1* status in FSHD2) and its peripheral subnuclear localization. Taken together our results detail the spatial organization of the 4q35 locus within the nucleus and reveal the existence of new topological domains that depend on the residual number of D4Z4 macrosatellite repeats (Fig. 3B,C).

### Changes in chromatin organization of the 4q35 locus correlate with modulation in gene expression

**Figure 4:**
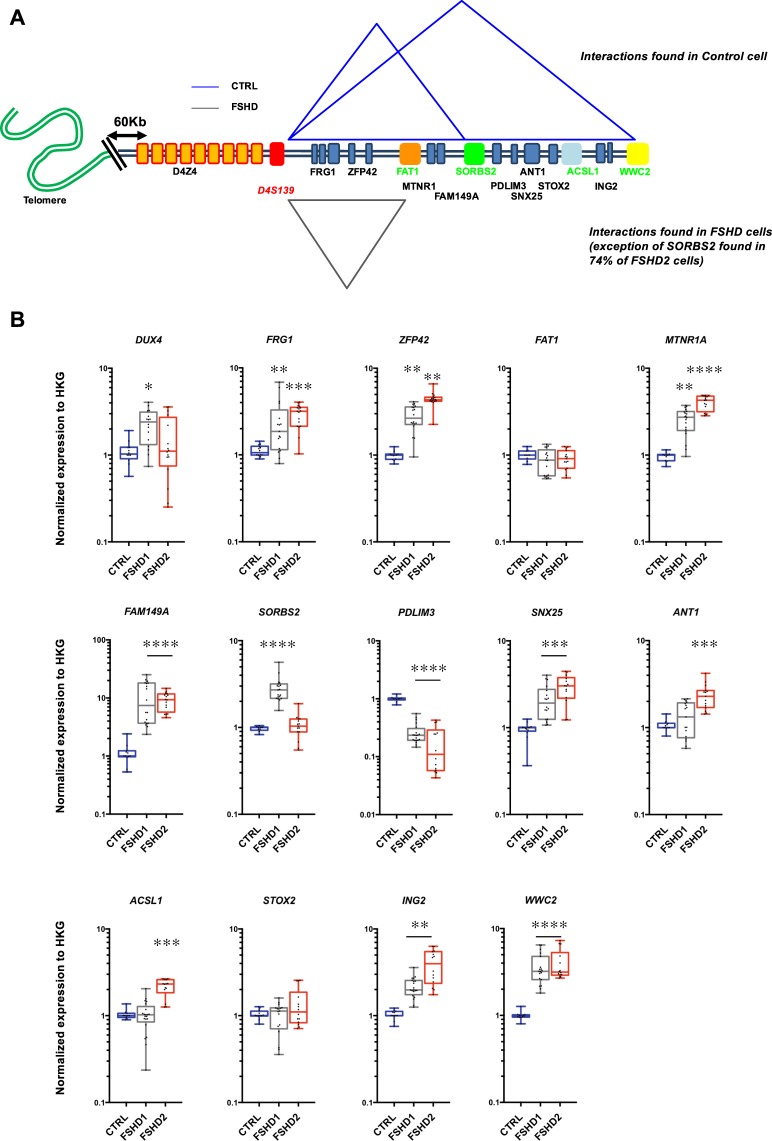
3D genome organization within the 4q35 locus and gene expression in FSHD cells (fibroblasts). Schematic representation of interactions described by 3D FISH experiments (**A**) and associated gene expression (**B**). Gene expression quantified by RT-qPCR in control (n = 3), FSHD1 (n = 5) and FSHD2 (n = 2) cells. Each measure represents the average fold-change expression of six independent assays (biological triplicate in technical RT duplicate) normalized to three housekeeping genes (HKG: *HPRT*, *PPIA* and *GAPDH*; ΔΔCt method). Differences compared to the control cells are represented as boxplot with associated statistical significance (Kruskal-Wallis multiple comparison test; α = 0.05) along with means ± SEM. *p < 0.05; **p < 0.01; ***p < 0.005; ****p < 0.001.

To further investigate the consequences triggered by changes in DNA folding within the 4q35 region, we examined the level of expression of different genes located within or outside the modulated chromatin loops by RT-qPCR (Fig. 4). Hence, we exploited a restricted gene expression analysis to decipher the emergence of additional territories by analyzing expression of 14 genes located at the 4q35 locus in our fibroblasts (Fig. 4). In our cellular model, we first determined the level of transcription as the ratio between the gene of interest and housekeeping genes for normalization (Supplementary Fig. 6). Next, we analyzed genes localized in loops present in control cells (Fig. 4A) but absent in FSHD cells and *vice versa*. Interestingly, expression of most genes within the last 7 Mb of chromosome 4q was significantly modulated (global upregulation; Supplementary Fig. 6, Fig. 4B; *e.g*., excluding *FAT1* and *STOX2*, *p* > 0.05; Kruskal-Wallis multiple comparison test; α = 0.05), with a high variability between samples, including between individuals with the same genotype (Fig. 4B).

First, considering the D4S139-*FAT1* loop (Fig. 4A), we detected a significant upregulation of the *FRG1* and *ZFP42* genes in all FSHD cells regardless of the D4Z4 status (FSHD1 and FSHD2) (*p* < 0.05; Kruskal-Wallis multiple comparison test; α = 0.05, Fig. 4B). *DUX4* was significantly upregulated only in FSHD1 fibroblasts (*p* = 0.0057; Kruskal-Wallis multiple comparison test; α = 0.05) compared to control cells, but not detectable in the FSHD2 cells tested here.

Next, given the strong interactions involving the D4S139 probe and other loci (*FAT1*, *SORBS2*; Figs 2 and 3), we deduced a global interaction between the three regions. Indeed, we found an interaction between D4S139 and *FAT1* for both alleles in most FSHD cells (±77.5%; Fig. 2) and between D4S139 and *SORBS2* for one allele in FSHD1 cells (±90%) and both alleles in most FSHD2 cells (76%; Fig. 3). This highly suggests an interaction between *SORBS2* and *FAT1* in FSHD2 cells and an interaction involving the healthy non-contracted allele in FSHD1 cells. Expression of genes located within this newly formed loop was significantly upregulated not only in FSHD cells presenting the interaction but across all samples (*MTNR1A*, *FAM149A*). This suggest other modulators of expression, including a possible unidentified loop^47^. Strikingly, expression of *SORBS2* was directly correlated to the formation of the loop. Only cells where the D4S139-*SORBS2* interaction was not detected presented upregulation of its expression (FSHD1; *p* < 0.001; Kruskal-Wallis multiple comparison test; α = 0.05, Fig. 4B). Of note, the *PDLIM3* gene, which shares a bidirectional promoter with *SORBS2*, displayed a downregulated expression in all FSHD cells.

Our set of 3D FISH assay did not allow us to detect other chromatin structures beyond the *SORBS2* locus in FSHD cells, whereas control cells showed a D4S139-*WWC2* interaction. Expression of genes beyond the *SORBS2* locus displayed mixed results. The *ING2* and *WWC2* gene were upregulated in FSHD cells (*p* < 0.001; Kruskal-Wallis multiple comparison test; α = 0.05) while *ANT1* and *ACSL1* were upregulated only in FSHD2 cells. Altogether, we found that in FSHD cells, levels of expression were globally increased beyond the *SORBS2* locus, advocating for a looser chromatin structure possibly permissive for transcription.

### 4q35 genes are differentially expressed in muscle biopsies

**Figure 5:**
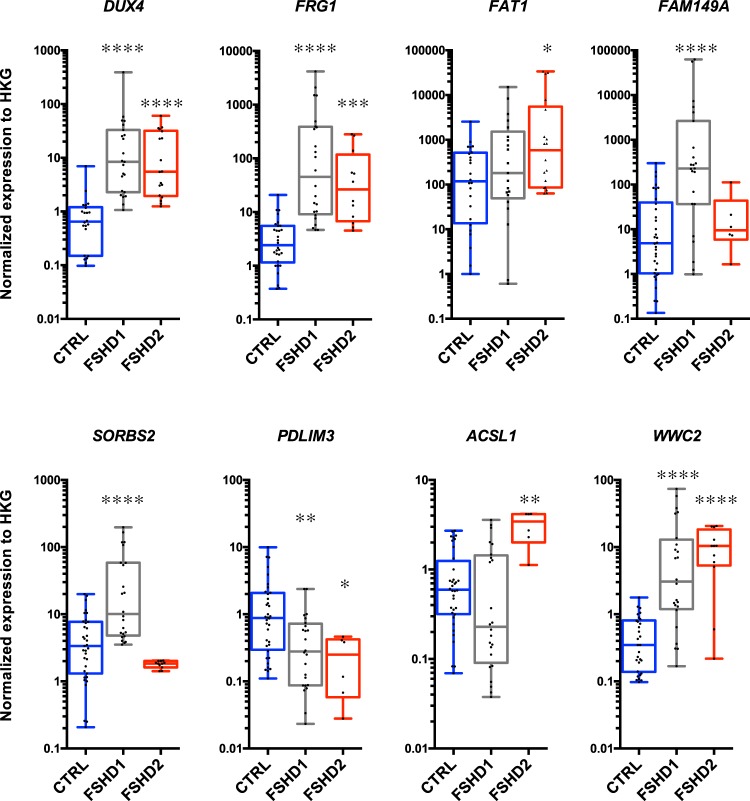
Expression of genes of the 4q35 region in controls and FSHD biopsies (muscle). Gene expression quantified by RT-qPCR in control (n = 7, Blue); FSHD1 (n = 7, Grey) and FSHD2 (n = 3, Red) muscle biopsies (*quadriceps femoris*). Each measure represents the average fold-change expression of six independent assays (RT triplicate, technical duplicate) normalized to three housekeeping genes (HKG: *HPRT*, *PPIA* and *GAPDH*; ΔΔCt method). Differences from the control group are represented as boxplots with associated statistical significance (Kruskal-Wallis multiple comparison test; α = 0.05) along with means ± SEM. *p < 0.05; **p < 0.01; ***p < 0.005; ****p < 0.001.

In primary fibroblasts, we found that the modification of the 4q35 chromatin landscape correlates with changes in transcription of genes located within the modified domains. In a second step, we thus consolidated our findings by analyzing gene expression directly in muscle biopsies isolated from individuals diagnosed with either FSHD1 or FSHD2. Hence, for confronting our *in vitro* data in primary fibroblasts with tissues affected in this pathology (*i.e*., skeletal muscle), we used a collection of biopsies from control and affected individuals (FSHD1, n = 7; FSHD2, n = 3; Control, n = 7; Table 2). As found by others, *DUX4* and *FRG1* expression were upregulated in FSHD biopsies (Fig. 5; *p* < 0.005; Kruskal-Wallis multiple comparison test; α = 0.05)^27,48,49^. Additionally, *FAM149A* and *SORBS2* were exclusively upregulated in FSHD1 (*p* < 0.001, respectively; Kruskal-Wallis multiple comparison test; α = 0.05); *FAT1* and *ASCL1* in FSHD2 (*p* = 0.023 and *p* = 0.006, respectively; Kruskal-Wallis multiple comparison test; α = 0.05).

**Table 2:** Summary of muscle biopsies used in the study with their respective status.

Sample	Status	Origin	Age	Sex
1	Control	Quad	77	♂
2	FSHD1	Quad	Fetus	♀
3	Control	Quad	Fetus	♂
4	Control	Quad	Fetus	♂
5	FSHD1	Quad	61	♂
6	FSHD2	Quad	57	♂
7	FSHD2	Quad	45	♂
8	FSHD2	Quad	42	♂
9	FSHD1	Quad	54	♂
10	Control	Quad	52	♂
11	Control	Quad	Fetus	♂
12	FSHD1	Quad	61	♂
13	Control	Quad	69	♂
14	Control	Quad	71	♀
15	FSHD1	Quad	Fetus	♂
16	FSHD1	Quad	Fetus	♀
17	FSHD1	Quad	Fetus	♀

FSHD individuals were first diagnosed by a clinical examination (except for fetuses obtained after medical abortion) and further confirmed by genetic testing (Southern Blot, DNA combing). Muscle origin, gender and age at biopsy are reported.

If one considers that chromatin territories are conserved among cell types, our results strongly suggest a direct correlation between 3D organization of the 4q35 locus (*e.g*., no interaction in FSHD1) and *SORBS2* or *WWC2* expression in FSHD. Moreover, the intriguing upregulated expression of *ASCL1* and *FAT1* restricted to FSHD2 biopsies advocates for a more complex regulation possibly involving SMCHD1 as described for other genomic regions^33,50^, or the existence additional DNA interaction yet to be characterized^16^.

### The 4q35 chromatin organization is set to a new conformation in hIPSC

**Figure 6:**
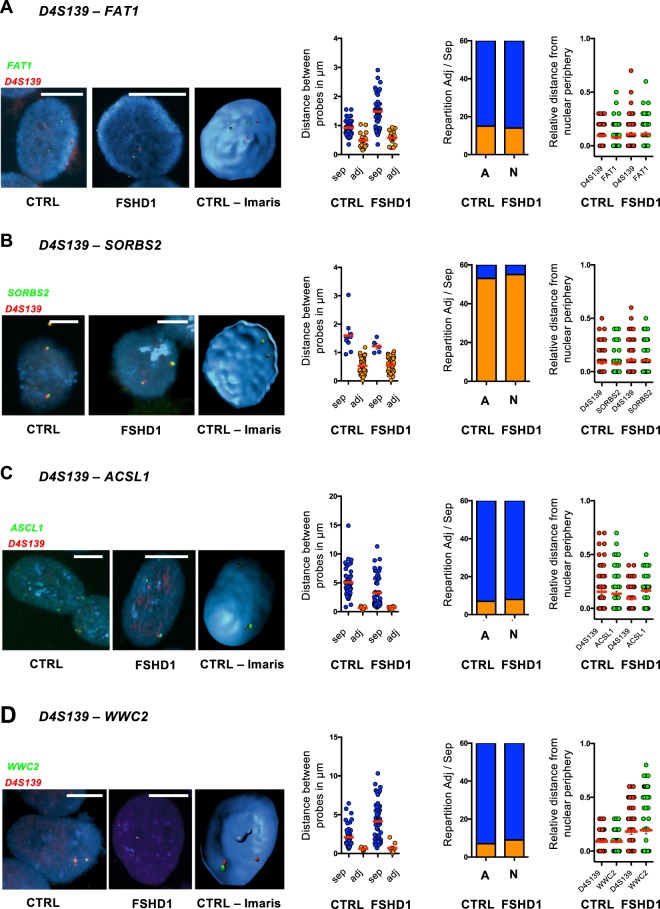
Topology of the 4q35 region is identical in control and FSHD pluripotent stem cells. Three-Dimension DNA FISH assay within the 4q35 locus in human Induced Pluripotent Stem Cells (hIPSC) generated from control and FSHD1 primary fibroblasts (**A**, N; respectively - see Table 1). 3D FISH was done using two sets of probes corresponding either to the D4S139 region (red) or different loci along the 4q35 chromosome (green*, FAT1*, *SORBS2*, *ASCL1* or *WWC2*; respectively). Representative pictures and 3D reconstruction using IMARIS along with their associated quantifications are presented. We measured the distance of each probe to the nuclear periphery and distance between the probes. For each cell, we evaluated if signals are separated or adjacent (sep, adj; respectively). Scattergram distribution of events and associated Chi-Square test (n = 30 cells per sample, 60 alleles) are shown. Scale is indicated by a white bar (=4 µm). No statistical differences were found between cells. (**A**) Localization between the D4S139 region and the *FAT1* gene. (**B**) Localization between the D4S139 region and the *SORBS2* gene. (**C**) Localization between the D4S139 region and the *ACSL1* gene. (**D**) Localization between the D4S139 region and the *WWC2* gene.

**Figure 7:**
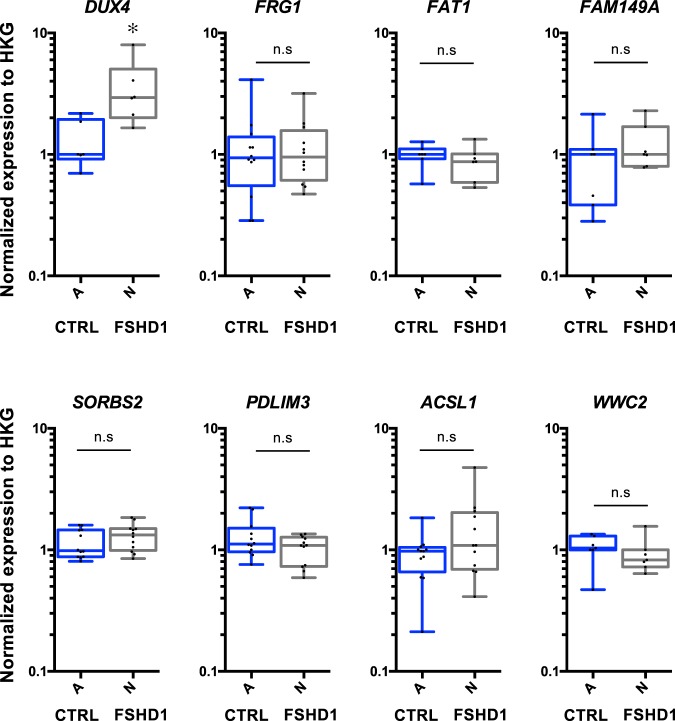
4q35 gene expression is not modulated in FSHD hiPSCs compared to controls. Gene expression quantified by RT-qPCR in control FSHD1 hIPSC (**A**, N; respectively - see Table 1). Each measure represents the average fold-change expression of six independent assays (Biological triplicate in technical RT duplicate) normalized to three housekeeping genes (HKG: *HPRT*, *PPIA* and *GAPDH*; △△Ct method). Differences relative to control cells are represented as boxplots with associated statistical significance (Mann Whitney; α = 0.05) along with Means ± SEM. *p < 0.05.

Earlier works have shown that the position of single gene loci and entire chromosomes is heritable after cell division^51^. TADs are largely maintained through development as TAD boundaries tend to be 65% similar among cell types^42,52,53^. However, HiC data revealed massive transformation in chromosome topology after reprogramming with many interactions present in the cells of origin being progressively erased from early to late passage^54^. Furthermore, the nuclear lamina undergoes profound changes since A-type Lamins present in the somatic cell-of-origin are not expressed in pluripotent cells^55^. Our recent findings showed that D4Z4 methylation is increased upon reprogramming in cells from FSHD1 patients^56^ suggesting chromatin changes at the 4q35 locus in pluripotent cells. We therefore investigated whether chromatin folding and 3D organization was inherited or modified after reprogramming by taking advantage of the chromatin organization in FSHD1 cells carrying both a contracted and non-contracted *D4Z4* allele (N, Table 1), thus displaying the 3D organization found across all FSHD cells (FSHD1 and FSHD2). We restrained our 3D DNA FISH analysis to hIPSCs generated from the corresponding fibroblasts (A and N; respectively). HIPSCs cells were characterized and described elsewhere^56,57^. We analyzed the 4q35 conformation using probes for the D4S139 region together with probes targeting the *FAT1*, *SORBS2*, *ACSL1* and *WWC2* loci (Fig. 6). Remarkably, no difference was found between control and FSHD1 hIPSCs. Signals were all localized at the nuclear periphery and only one long-distance interaction was preserved after reprogramming between D4S139 and *SORBS2* (Fig. 6B), consistent with the main TAD found in other cell types (Supplementary Fig. 1). Indeed, the longest interaction observed in control cells encompassing D4S139 and the *WWC2* region (7 Mb) was lost in all hIPSCs. This observation suggests a loop established *de novo* in control but not FSHD cells, reinforcing the hypothesis of a disturbed development in FSHD cells^48,58,59^. In parallel, we tested gene expression by RT-qPCRs (Fig. 7). Our data revealed no significant difference between conditions, expect for the *DUX4* gene upregulated in FSHD1 hiPSCs (*p* = 0.0130; Mann Whitney test; α = 0.05).

In conclusion, as seen by others, *DUX4* expression was independent of the centromeric 4q35 chromatin organization, but might be under the influence of other chromatin features such as changes in DNA methylation, attachment to the nuclear matrix or telomere length^28,29,37^. Overall, our data suggest that the 4q35 3D organization is modified in differentiated cells (fibroblasts) in pathological conditions, *i.e*., upon shortening of the D4Z4 array or in cells carrying a mutation in *SMCHD1*. At the pluripotent state, cells display a domain encompassing the most subtelomeric part of the locus and the *SORBS2* region, which appears to be the most conserved domain among cell types as also revealed by HiC data available in the literature^6,42^ (Supplementary Fig. 1). Thus, upon reprogramming, cells undergo a profound reorganization of the 4q35 chromatin structure with an increased level of methylation in FSHD1 cells^56^ but also, as shown here, erasure of long-distance interactions indicating a dynamic organization of the 4q35 locus depending on the number of D4Z4 macrosatellite repeats and its epigenetic status.

## Discussion

In this study, we investigated the organization of the 4q35 locus linked to Facio-Scapulo Humeral Dystrophy in different contexts. Overall, our data show important 3D changes within the 4q35 locus due but not limited to D4Z4 array contraction. We observed differences in chromatin organization between cells from controls and individuals with clinical signs of the disease (*e.g*., FSHD1, FSHD2). While one interaction was dependent of the D4Z4 array (*e.g*., D4S139-*SORBS2*), most interactions are independent of the number of D4Z4 repeats (FSHD2 cases, Figs 2 and 3). This chromatin landscape is profoundly modified upon reprogramming with erasure of the parent-of-origin architectural organization and establishment of a novel pluripotent-specific profile identical between controls and FSHD1 cells (Fig. 6). In somatic cells, modified long-distance interactions correlate with different expression signatures. This observation was validated in muscle biopsies (Fig. 5) and advocates for a conserved topology among cell types and a role for D4Z4 or SMCHD1 in this higher-order organization, with consequences on gene expression. We also observed that expression of *DUX4* was independent of changes occurring in the chromatin organization of more centromeric territories, hence confirming findings of others^28–30,35,36^.

Importantly, our work based on confocal imaging, which provides chromatin analysis at the single cell and single allele level provides a precise representation and frequency of events across multiple samples and for each individual allele within a cell. This technical design limits overrepresentation of discrete events that might bias data interpretation as sometimes observed using 3C techniques^36^. Likewise, previous work based on DNA replication timing experiments revealed high similarities between samples (97.3%) and cell types (>80%) without any difference for the 4q35 locus between samples^60^. Those conflicting observations (interactions *vs*. no interactions) could arise from difficulties inherent to the analysis of subtelomeric regions in general (*i.e*., GC-rich, late replication), insufficient depth of sequencing and incomplete assembly of subtelomeric sequences^61–63^. Altogether, these works using sequencing techniques highlight the challenge in analyzing the 4q35 region as illustrated by our recent findings describing complex *cis* rearrangements in a subset of patients^45^ and raise further questions on the peculiar organization of the 4q35 loci in FSHD, where one needs to consider the specificity of each allele. By using only samples from well characterized cells with regard to number of repeats (analyzed by Southern blot or DNA combing)^45^, these concerns were carefully considered in the design of our study.

Boundary elements or insulators are critical for the establishment or maintenance of the genome architecture. Boundary elements bound by the CTCF protein concur to the formation of TADs and mediate long-distance interactions. At the 4q35 region, we have described D4Z4 as a genomic element acting as a CTCF and A-type Lamins dependent insulator^13^ and able to tether a telomere at the nuclear periphery^12^. We have also identified additional sequences with insulator activity along the 4q35 region, which are not able to tether the 4q35 to the nuclear periphery (Morere and Magdinier, unpublished data). This indicates a key role for D4Z4 in the positioning of this region within the nuclear space but also in the establishment of long distance interactions. In agreement with this hypothesis, we previously showed that in the context of a short D4Z4 array and short telomeres, disruption of 4q35 chromatin loops modulate gene expression but more strikingly splicing of genes such as *SORBS2* or *FAM149A*^16^. Using different cells, our present work confirms the existence of these interactions (Figs 3, 5) with a loop encompassing the *SORBS2* region and the D4Z4 array, conserved in cells with long D4Z4 array. Interestingly, this loop loosened for one out of the two alleles in FSHD1 fibroblasts is reset in hiPSCs after reprogramming and telomerase reactivation. Strikingly, the other 3D structures were completely lost (*e.g*., D4S139-*FAT1* restricted to FSHD*;* D4S139-*WWC2* limited to controls) reinforcing the importance of the telomere vicinity in the regulation of this locus^12,16,23^. Interestingly, these loops do not depend on *SMCHD1* gene dosage, since long distance interactions are maintained in most FSHD2 cells (Fig. 3B; 76%) carrying a heterozygote truncating mutation. This further argues in favor of a pleiotropic role for this chromatin factor with a role in D4Z4 regulation which likely differs from its topological role in the regulation of *Hox* genes or formation of A and B compartments in X inactivation in the mouse^33,50^.

At the 4q35 locus, the territory identified by 3D FISH is reminiscent of the TAD identified in different cell types^6^, advocating for a pivotal role in the setting of boundaries within the 4q35 region. Further, by investigating hIPSCs which do not express A-type Lamins^55^, we observed that FSHD1 cells did not exhibit the same chromatin organization as somatic cells of origin with a loss in the 3D Chromatin structure present before reprograming. This intriguing result reveals that tethering to the nuclear periphery of the 4q35 region is not alleviated by absence of A-type Lamins as previously observed using artificially fragmented telomeres^12^. In this telomerase positive context, the short D4Z4 array (*e.g*., exhibiting an un-masked CTCF site) harbors a peripheral nuclear position but could be untangled with an abutting long telomere, hence masking the CTCF site and modification of long distance interactions. The total topological re-set towards a new organization common between controls and diseased cells suggests a model where differences in chromatin topology would arise during or after differentiation. Hence, one could hypothesize that the FSHD chromatin landscape is not inherited but progressively installed upon differentiation.

Based on our observations, we propose here a complex orchestration of the 4q35 locus folding upon differentiation from a pluripotent state (*i.e*., hIPSC) with clear differences between control cells and cells from patients affected with FSHD. Combined with previous findings^12,13,16,23^, defining the dynamic 3D organization of this locus in different genetic contexts will provide further insights into the understanding of its regulation and its impact on the transcriptional signature of 4q35 genes during development and differentiation towards the muscle lineage. Furthermore, our results confirm a role of the D4Z4 macrosatellite in the organization of chromatin in human cells and formation of long-distance loops.

## Materials and Methods

The present study, protocols and experiments were approved, performed and validated following the scientific guidelines provided by the Agence National pour la Recherche (ANR); INSERM (U1251 laboratory) and AFM. Research was approved by the biomedicine agency (PFS13-006).

### Study samples

All individuals have provided written informed consent for the collection of samples and subsequent analysis for medical research. The study was done in accordance with the Declaration of Helsinki and samples were obtained from the Centre de Ressources Biologiques (CRB) Hôpital de la Timone Enfant, Marseille (CRB AP-HM 0COPE05E001). Controls are randomly selected individuals or patient’s relatives, selected in the same age range and sex representation as patients. Controls are neither carrier of any genetic mutation nor affected by any constitutive pathology. Samples are listed in Tables 1 and 2.

### Reprogramming of human fibroblasts and production of hiPSCs

Human iPSCs were generated as described elsewhere^57,58^. HiPSCs colonies were picked about 4 to 6 weeks after reprogramming based on their ES cell-like morphology. Colonies were grown and expanded in mTeSR™1 medium (StemCells) on dishes coated with Matrigel™ (Corning, cat, No. 354277). The different clones used were fully characterized using classical procedures^56,57^.

### RNA extraction and quality control

Total RNA was extracted using the RNAeasy kit (Qiagen) following manufacturer’s instructions. Quality, quantification and sizing of total RNA was evaluated using the RNA 6000 Pico assay (Agilent Technologies Ref. 5067-1513) on an Agilent 2100 Bioanalyzer system.

### Quantitative RT-PCR

Reverse transcription of 500 ng of total RNA was performed using the Superscript II or IV kit and a mixture of random hexamers and oligo dT following manufacturer’s instructions at 42 °C for 50 minutes followed by inactivation at 70 °C for 15 minutes (Life Technologies). Primers were designed using Primer Blast and Primer3 (See list attached below). Real-time PCR amplification was performed on a LightCycler 480 (Roche) using the SYBR green master mix. All PCR were performed using a standardized protocol and data were analyzed with the Lightcycler 480 software version 1.5.0.39 (Roche). Primer efficiency was determined by absolute quantification using a standard curve. For each sample, fold-change was obtained by comparative quantification and normalization to expression of the *HPRT, GAPDH and PPIA* (*DUX4* and 4q35 genes) housekeeping genes used as standard. Data are expressed as means ± SEM.

**Table:** 

Gene	Forward Primer	Reverse Primer
*HPRT*	tgatagatccattcctatgactgtaga	caagacattctttccagttaaagttg
*GAPDH*	agccacatcgctcagacac	gcccaatacgaccaaatcc
*PPIA*	atgctggacccaacacaaat	tctttcactttgccaaacacc
*DUX4*	cccaggtaccagcagacc	tccaggagatgtaactctaatcca
*FRG1*	ttgttggaatctggtggaca	gcccaacaacaagtccatct
*FAT1*	atgggaggtcgattcacg	cattagagatggctctggcg
*FAM149A*	ccgccccagattcatcactt	gctgaagcggttttgcttga
*SORBS2*	aacctttaaagcaagaggaggcgag	tttcccctgtagcctagctcagc
*PDLIM3*	ggtgctgtggtgaaggcgcg	gccctctgggggctttgtgc
*ACSL1*	cctgggattcacttctccaggga	gtgcaagccctgttgtgcttgt
*WWC2*	tgacaatatggcagttcgcccca	tcactgtcactccgatttaacctgc

### 3D Fish

Cells were grown on 4-well slides (Millipore) or 4-well PCA slides (hIPSCs; Sarstedt), fixed in 4% paraformaldehyde and treated as described^12^ to maintain the 3D structure of the cells. All probes were denatured at 80 ± 1 °C for 5 minutes before hybridization. Nuclei were counterstained with DAPI (Sigma) diluted to 1 mg/ml in PBS and mounted in Vectashield (Vector Laboratories). Probes were either purchased (RainbowFISH, Cytocell) or produced by nick translation using after-mentioned BAC clones (CHORI) as template: RP11-521G19 (D4S139); RP11-33M11 (*FAT1*); RP11-159A22 (*SORBS2*); RP11-374K13 (*ASCL1*) and RP11-451F20 (*WWC2*), respectively.

Images were acquired using a confocal scanning laser system from Zeiss (Germany). A 63x Plan-APOCHROMAT, oil immersion, NA 1.40 objective (Zeiss) was used to record optical sections at intervals of 0.24 µm. The pinhole was set the closest to 1 Airy with optical slices in all wavelengths with identical thickness (0.4 µm). Generated.lsm files with a voxel size of 0.1 µm × 0.1 µm × 0.24 µm were processed using the IMARIS software (Bitplane, AG). After 3D analysis, we compared the mean volume ratio of nuclei and of the probe signals. Values were compared using a non-parametric Kruskal-Wallis test; population of separated or adjacent signals were compared to the control condition using a Chi-square test.

## Supplementary information

Supplemental Figures
