# Supplementary material for: Analysis of the 4q35 chromatin organization reveals distinct long-range interactions in patients affected with Facio-Scapulo-Humeral Dystrophy
Source: Sci Rep. 2019 Jul 17;9:10327. doi: 10.1038/s41598-019-46861-x (PMC6637155; doi:10.1038/s41598-019-46861-x)
Supplement: Supplementary file 1 — Supplemental Figures [file 41598_2019_46861_MOESM1_ESM.pdf]

Analysis of the 4q35 chromatin organization reveals distinct long-range interactions in patients affected with Facio-Scapulo-Humeral Dystrophy.

Marie-Cécile Gaillard[1*], Natacha Broucqsault[1*], Julia Morere[1], Camille Laberthonnière[1], Camille Dion[1], Cherif Badja[1], Stéphane Roche[1], Karine Nguyen[1,2], Frédérique Magdinier[1#*], Jérôme D. Robin[1#*].

Supplemental Figures

Supplemental Figure S1
- Chromatin conformation capture of the 3D organization of the 4q35 locus (last 7Mb).

Supplemental Figure S2
- Processing of 3D reconstructed nuclei.

Supplemental Figure S3
- 3D DNA FISH between the distal 4q35 region and the SORBS2 gene.

Supplemental Figure S4
- 3D DNA FISH between the distal 4q35 region and the ACSL1 gene.

Supplemental Figure S5
- 3D DNA FISH between the distal 4q35 region and the WWC2 gene.

Supplemental Figure S6
- Expression of genes of the 4q35 region in controls and FSHD cells.

Supplemental Figure S1

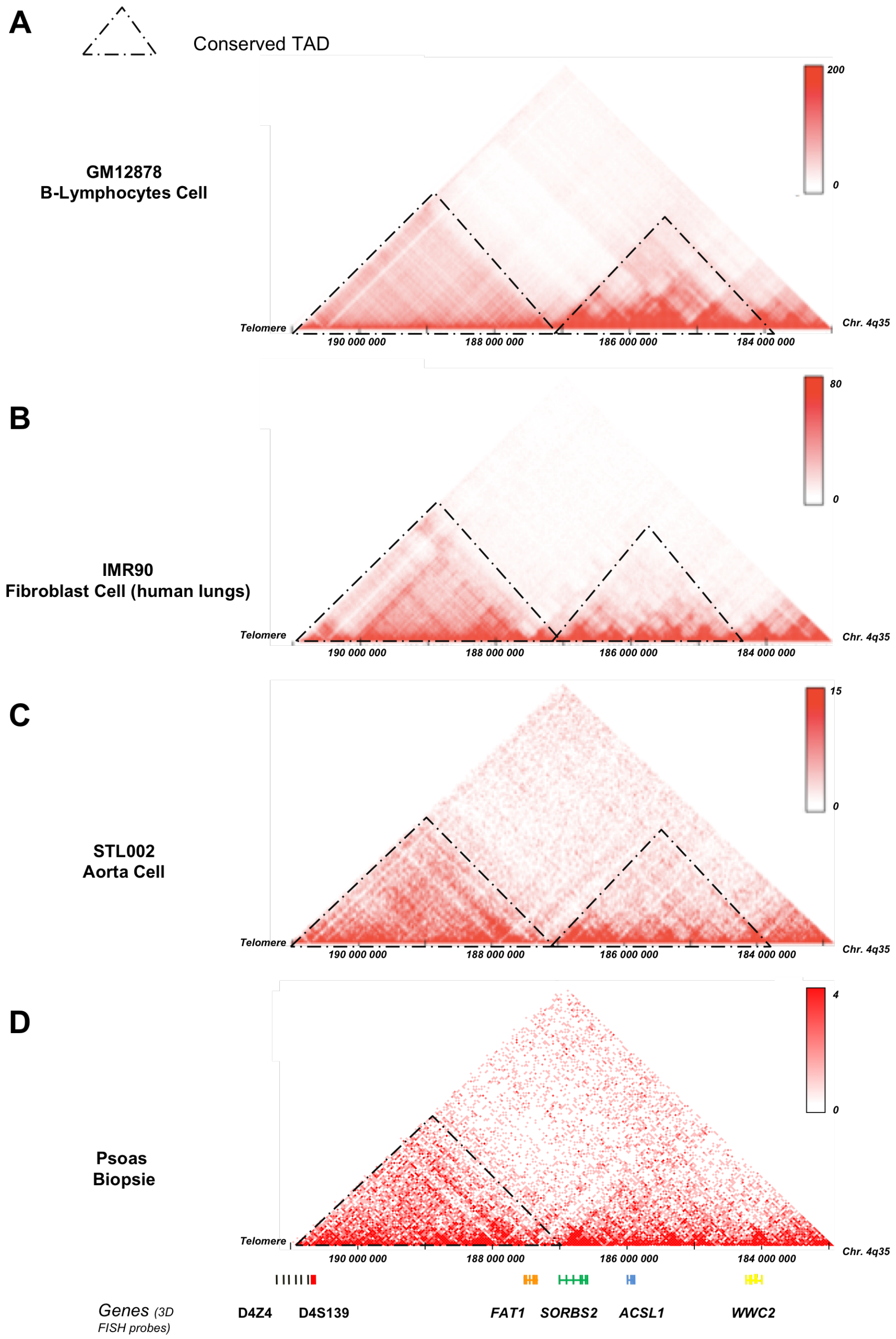

Supplementary figure 1. Chromatin conformation capture of the 3D organization of the 4q35 locus (last 7Mb).
Representative heat maps of the last 7Mb of the 4q35 locus, revealed in other studies by HiC or ChIA-PET (STL002) and available in the "3D Genome Browser" (ENCODE project 6). A. As shown by the scale intensity (200 and 80; respectively), we present the two heat maps with the highest resolution for Lymphocytes (GM12878) and fibroblasts (IMR90). B. Data from the most relevant tissue found in the database are also presented (*e.g.*, muscle related cells/biopsy). The two sets of data were generated from a ChIA-PET (STL002, aorta cells) and a HiC (Psoas muscle biopsy) assay with a low resolution (15 and 4; respectively). Each map is presented with the conserved Topologically Associated Domains (TADs). The most subtelomeric TAD (stopping between the *FAT1* and *SORBS2* region) is conserved between samples.

Supplemental Figure S2

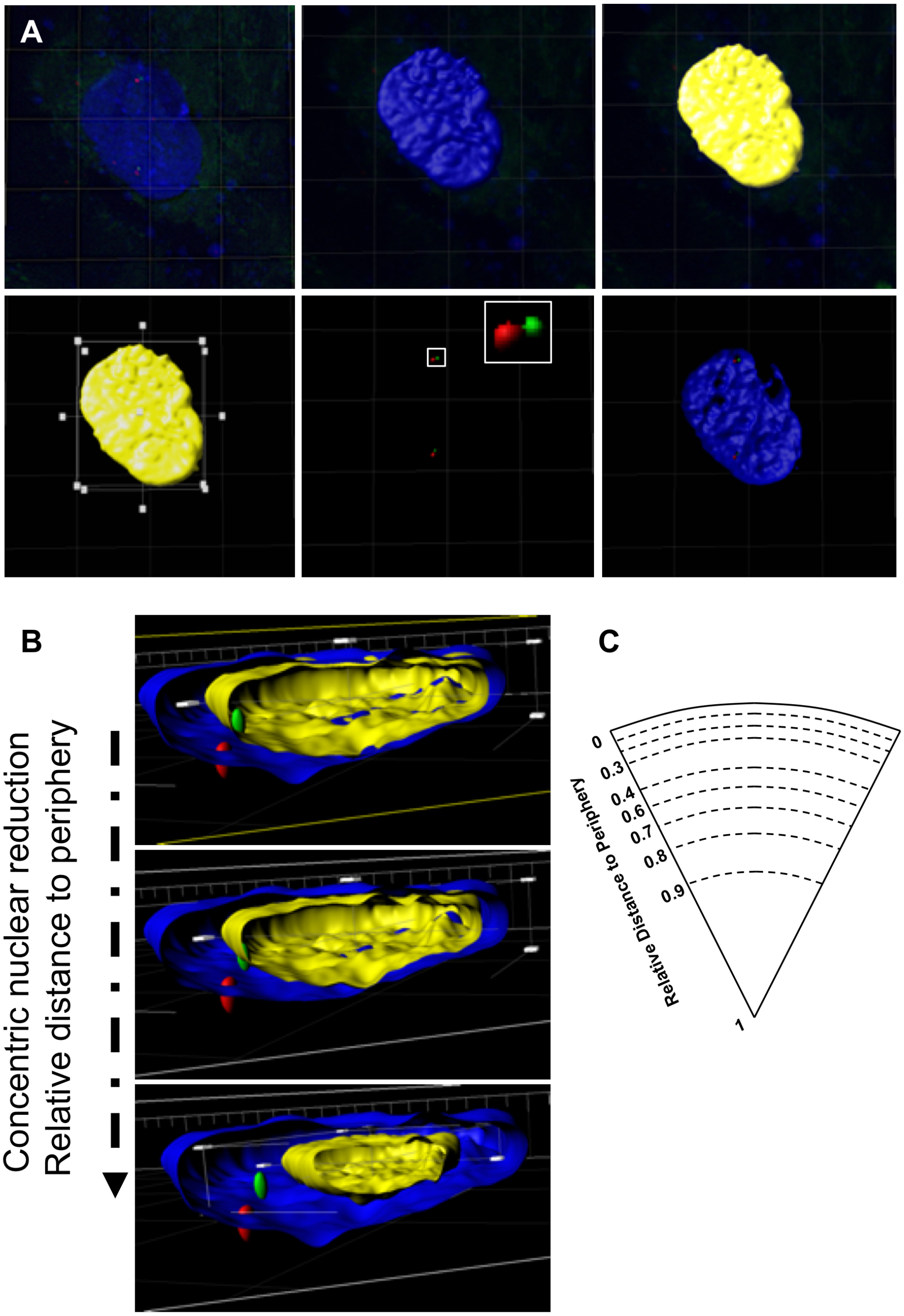

Supplementary figure 2. Processing of 3D reconstructed nuclei.

Nuclei with regular ellipsoidal shape were selected and each nucleus was analyzed independently. A. Thresholded DAPI and probe signals were acquired by confocal microscopy (z-stacks of 0.24µm thickness). Three-dimensional objects were created for each. B. A reduced reproduction (yellow structure) of the object corresponding to the nuclear rim (Blue) was generated having the same center defined as the center of the cuboid in which that object is inscribed, with dimensions reduced until the FISH signal matches the reduced envelope. C. For each nucleus, this reduction of dimensions was mathematically turned into equivalent sphere volume reduction in order to obtain a distribution of the FISH signal within the nuclear volume for each experimental condition.

Supplemental Figure S3

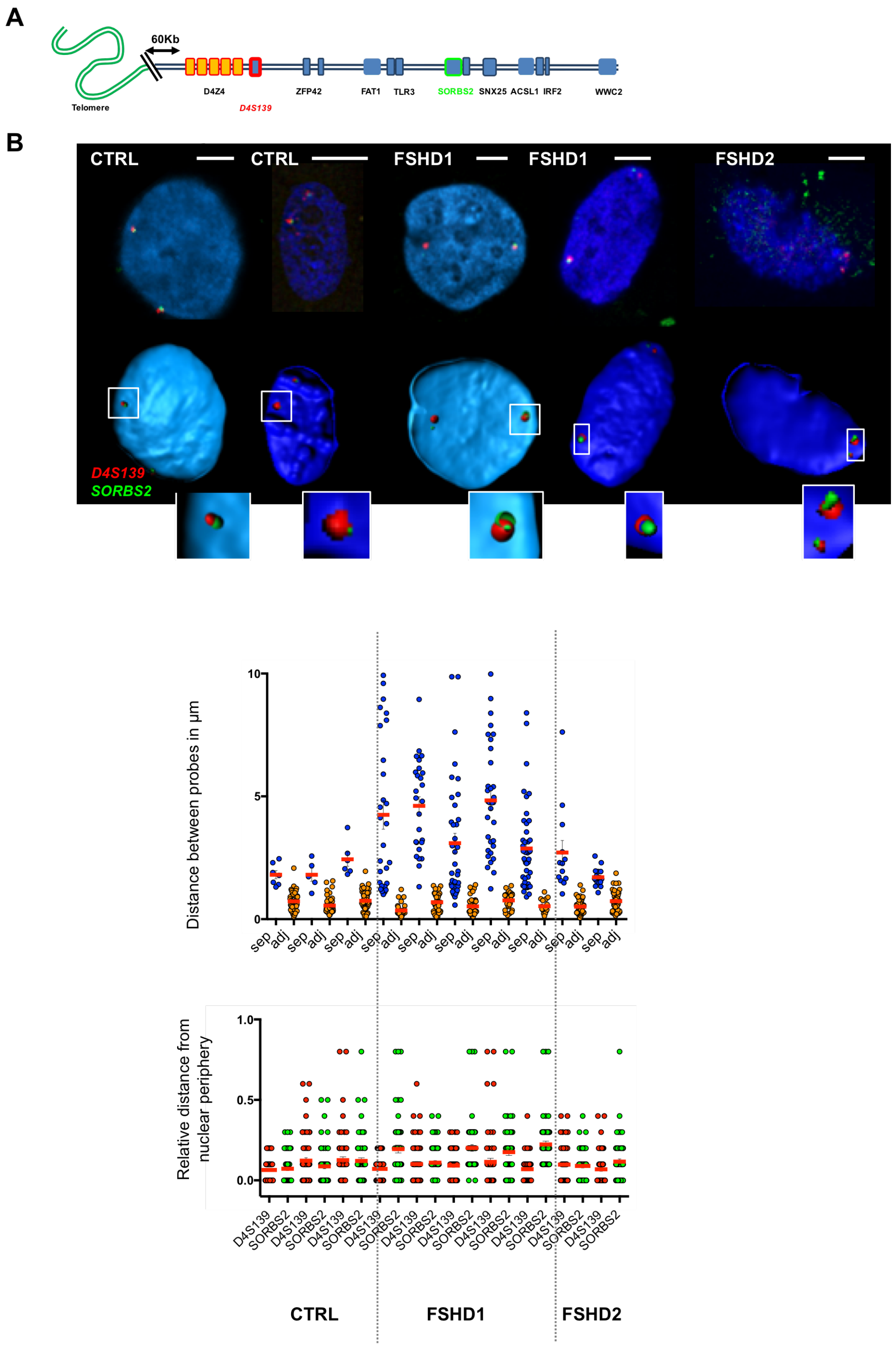

Supplementary figure 3. 3D DNA FISH between the distal 4q35 region and the *SORBS2* gene. A. Graphical representation of the last 7Mb of the chromosome 4q arm, from the telomere (green) to the *WWC2* gene (blue square). 3D FISH was done using two sets of probes corresponding either the D4S139 VNTR (red) or the *SORBS2* region (green). **B.** Representative pictures and 3D reconstruction using IMARIS are presented along with their associated quantifications. Scale is indicated by a white bar (=2µm) **C.** We measured the distance of each probe to the nuclear periphery and distance between the probes. **D.** For each sample, histogram displays the percentage of separated or adjacent signals (sep, adj; respectively). Statistical significance was determined using a Chi-Square test (n=30 cells per sample, 60 alleles). **E.** Distribution of the average distances between probes and their respective distances to the periphery in each cell line. In one FSHD1 cell line (N), signals were adjacent for only one out of two probes (85% of cells), likely corresponding to the non-contracted allele. Frequencies of adjacent signals are decreased in all FSHD1 cells. ✻✻✻ $p < 0.005$; ✻✻✻✻ $p < 0.001$. Signals are mostly found at the periphery regardless of disease status.

Supplemental Figure S4

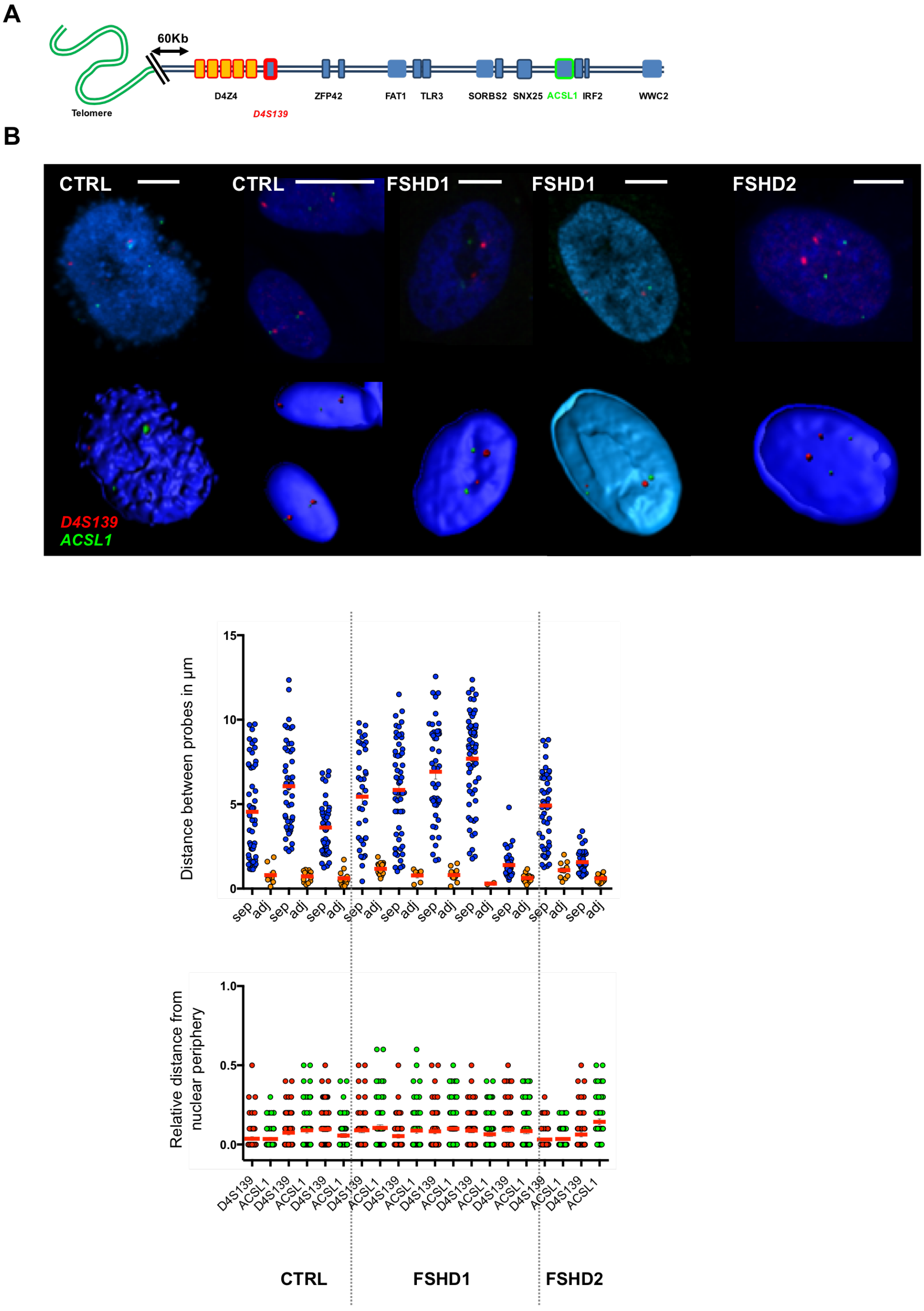

Supplementary figure 4. 3D DNA FISH between the distal 4q35 region and the *ACSL1* gene. A. Graphical representation of the last 7Mb of the chromosome 4 long arm, from the telomere (green) to the *WWC2* gene (blue square). 3D FISH was done using two sets of probes corresponding either the D4S139 region (red) or the *ASCL1* locus (green). **B.** Representative pictures and 3D reconstruction using IMARIS along with associated quantifications. Scale is indicated by a white bar (=2µm) **C.** We measured the distance of each probe to the nuclear periphery and distance between the probes. **D.** For each cell, we evaluated if signals were separated or adjacent (sep, adj; respectively). Statistical significance was determined using a Chi-Square test (n=30 cells per sample, 60 alleles). **E.** Graphical representation of the average distances between probes and their respective distances to the periphery in each cell line. Signals are mostly found at the periphery regardless of disease status. Localization between the different signals is not statistically different between samples.

Supplemental Figure S5

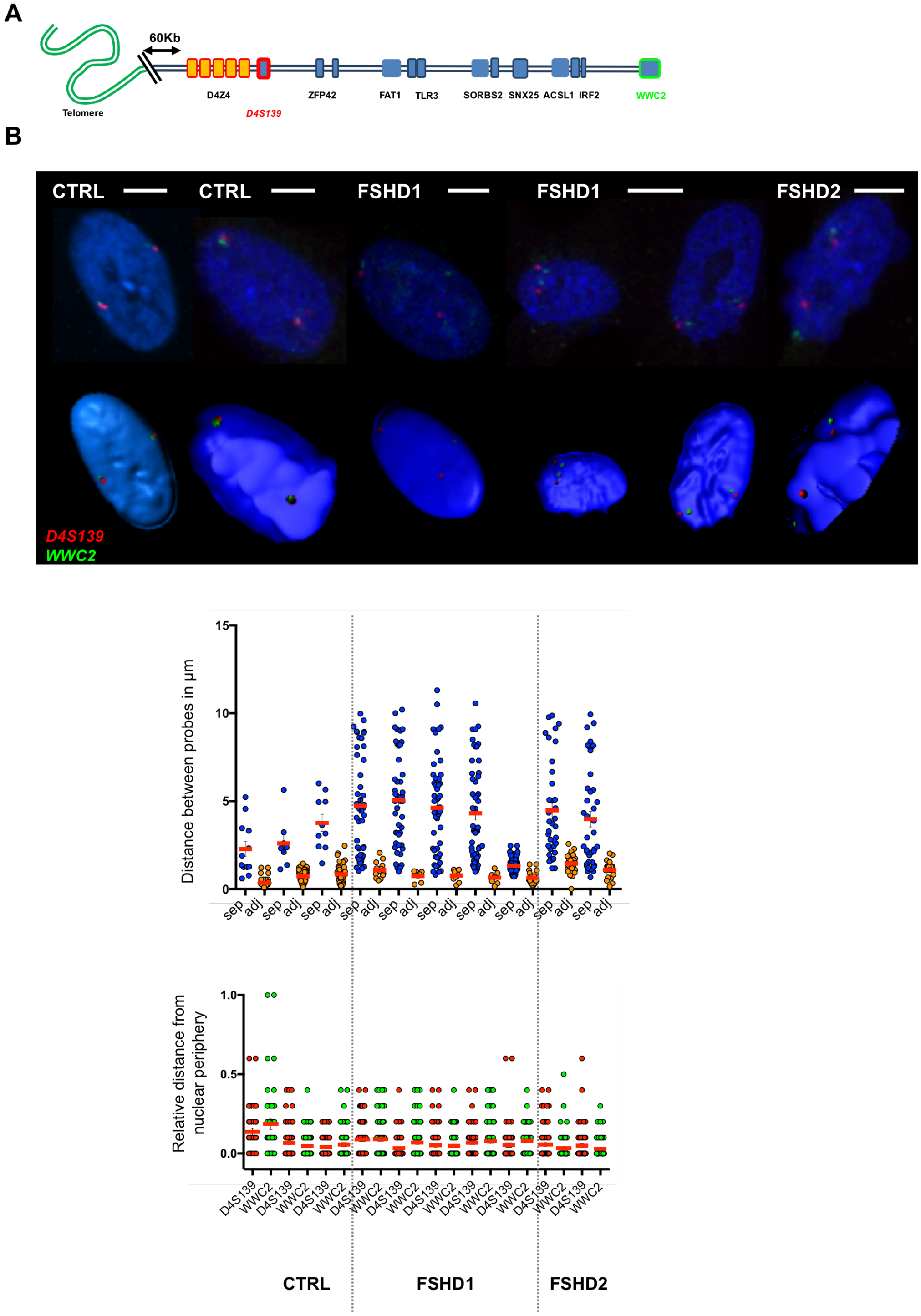

Supplementary figure 5. 3D DNA FISH between the distal 4q35 region and the *WWC2* gene. A. Graphical representation of the last 7Mb of the chromosome 4 long arm, from the telomere (green) to the *WWC2* gene (blue square). 3D FISH was done using two sets of probes corresponding either the D4S139 (red) or the *WWC2* region (green). **B.** Representative pictures and 3D reconstruction using IMARIS along with their associated quantifications. Scale is indicated by a white bar (=2µm) **C.** We measured the distance of each probe to the nuclear periphery and distance between the probes. **D.** For each cell, we evaluated if signals were separated or adjacent (sep, adj; respectively). Statistical significance was determined using a Chi-Square test (n=30 cells per sample, 60 alleles). **E.** Graphical abstract of the average distances between probes and their respective distances to the periphery in each cell type. Signals are mostly found at the periphery regardless of disease status. Frequencies of adjacent signals are decreased in all FSHD cells. ✻✻ $p < 0.01$; ✻✻✻✻ $p < 0.001$.

Supplemental Figure S6

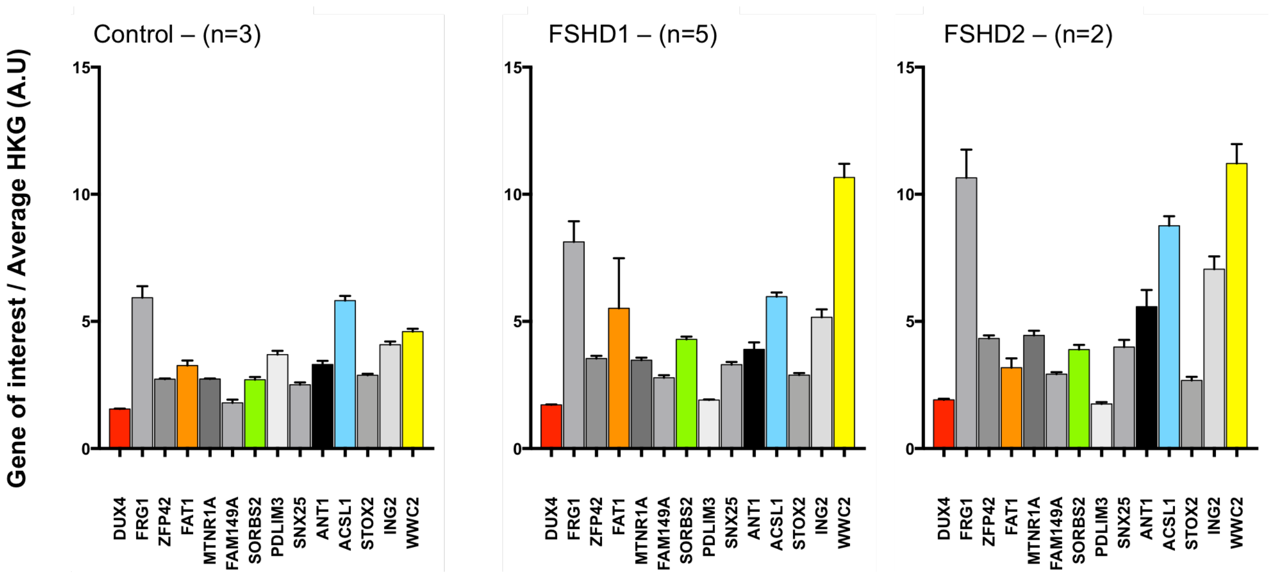

Supplementary figure 6. Expression of genes of the 4q35 region in controls and FSHD cells. We report the same RT-qPCR assay as in Supplementary Figure. 6 but using only normalization to House Keeping Genes (HKG: *HPRT*, *PPIA* and *GAPDH*; Gene/HKG ratio is represented) in control (n=3) FSHD1 (n=5) and FSHD2 (n=2) cells**.** Each measure represents the average fold-change expression of six independent assays (biological triplicate in technical RT duplicate). Ratios are reported per disease statuses and organized regarding to their genomic localization in order to reveal potential domains (as domain of expression ruled by similar epigenetic markers and chromatin organization).
